# Going Forward: Potential Impact of Protein-Based COVID-19 Vaccination Coverage on Population Outcomes and Costs in the United States

**DOI:** 10.3390/vaccines12010074

**Published:** 2024-01-12

**Authors:** Kyle Paret, Hadi Beyhaghi, William L. Herring, Josephine Mauskopf, Lesley G. Shane, Matthew D. Rousculp

**Affiliations:** 1 RTI Health Solutions, Research Triangle Park, NC 27709, USA; kparet@rti.org (K.P.); wherring@rti.org (W.L.H.); jmauskopf@rti.org (J.M.); 2 Novavax, Inc., Gaithersburg, MD 20878, USAlesleygshane@gmail.com (L.G.S.); 3 Karolinska Institute, 17177 Stockholm, Sweden

**Keywords:** COVID-19, vaccination, economic analysis, population health

## Abstract

Policymakers in the United States (US) recommend coronavirus disease 2019 (COVID-19) vaccination with a monovalent 2023–2024 vaccine formulation based on the Omicron XBB.1.5 variant. We estimated the potential US population-level health and economic impacts of increased COVID-19 vaccine coverage that might be expected with the availability of a protein-based vaccine with simpler storage requirements in addition to messenger ribonucleic acid (mRNA) vaccines. A Markov model was developed to estimate 1-year COVID-19-related costs, cases, hospitalizations, and deaths with and without the availability of a protein-based vaccine option. The model population was stratified by age and risk status. Model inputs were sourced from published literature or derived from publicly available data. Our model estimated that a five-percentage-point increase in coverage due to the availability of a protein-based vaccine option would prevent over 500,000 cases, 66,000 hospitalizations, and 3000 COVID-19-related deaths. These clinical outcomes translated to 42,000 quality-adjusted life years (QALYs) gained and an incremental cost–effectiveness ratio of USD 16,141/QALY from a third-party payer perspective. In sensitivity analyses, outcomes were most sensitive to COVID-19 incidence and severity across age groups. The availability of a protein-based vaccine option in the US could reduce hospitalizations and deaths and is predicted to be cost-effective.

## 1. Introduction

The coronavirus disease 2019 (COVID-19) pandemic was declared by the World Health Organization in 2020 in response to a new coronavirus (severe acute respiratory syndrome coronavirus 2 (SARS-CoV-2)) that spread aerobically and could cause symptoms of severe respiratory disease. As the virus spread, hospitals throughout the world were soon overwhelmed with infected individuals experiencing severe respiratory difficulties, often requiring mechanical ventilation to survive and suffering a high mortality rate [1]. As of the summer of 2023, the United States (US) had recorded at least 104 million cases of COVID-19, resulting in 6.1 million hospitalizations and 1.1 million deaths [2]. In addition to the devastating public health toll of COVID-19, far-reaching social and economic impacts of the pandemic were felt immediately [3] and have persisted [4].

This continued burden persisted even with effective vaccines against the original strain of the virus becoming widely available in early 2021 followed by monovalent and bivalent booster vaccines targeting later strains of the virus. As the SARS-CoV-2 virus continued to mutate and circulate in 2023, policymakers with the US Food and Drug Administration (FDA) advised manufacturers in June 2023 to develop updated monovalent COVID-19 vaccines based on the Omicron XBB.1.5 variant lineage for the 2023–2024 vaccination season [5]. In September 2023, the Centers for Disease Control and Prevention (CDC) issued updated COVID-19 vaccination guidance for the 2023–2024 season. The CDC recommended that all individuals aged 6 months and older be vaccinated with an updated monovalent 2023–2024 COVID-19 vaccine [6]. These vaccines include two updated vaccines from Pfizer/BioNTech and Moderna licensed by the FDA using the messenger ribonucleic acid (mRNA) technology for those aged 6 months and older [7] and an updated vaccine from Novavax using an adjuvanted protein-based technology with FDA emergency use authorization for those aged 12 years and older [8].

The shift toward what may become a regular annual cycle of COVID-19 vaccination in the US is taking place in a public health and reimbursement landscape that has changed markedly from earlier in the pandemic. The majority of the US population has been vaccinated previously with a primary COVID-19 vaccine series or has been infected with the SARS-CoV-2 virus (if not both) [1,9]. However, by the end of 2022, only 19.4% had subsequently received booster vaccination with a bivalent mRNA vaccine targeting two strains of the SARS-CoV-2 virus, resulting in uncertainty about the level of underlying immunity against current and future strains. Vaccine hesitancy [10,11] or difficulty storing the mRNA vaccines [12] may have contributed to the relatively low vaccine coverage in the US, especially in rural regions [9], compared with other high-income countries [13]. In addition, with the expiration of the federal COVID-19 public health emergency declaration in May 2023 [2], distribution and payment for the updated COVID-19 vaccines has moved from the US government, which purchased vaccines directly from manufacturers at negotiated prices, to traditional distribution systems and commercial healthcare payers.

Models developed for economic evaluations of interventions for SARS-CoV-2 initially focused on nonpharmaceutical initiatives to help with immediate maximization of resources [14]. Economic evaluations of COVID-19 vaccination programs conducted during the pandemic consistently supported the public health impact and the cost-effectiveness, or value for money, of vaccination against the SARS-CoV-2 virus [15,16]. As the pandemic has evolved toward a more endemic state, the needs of public health policymakers worldwide for COVID-19 economic evaluations are beginning to mirror other such vaccine-preventable diseases. For example, the Joint Committee on Vaccination and Immunisation in the United Kingdom made policy decisions about which risk groups to vaccinate with updated COVID-19 vaccines in the autumn of 2023 based on an economic model that estimated the population net benefits using estimates of the number needed to vaccinate to prevent different COVID-19 outcomes, recognizing the uncertainty of emerging mutations and their impact on vaccine effectiveness [17]. In the US, the Advisory Committee on Immunization Practices also included the results from an economic evaluation of the mRNA vaccines in their deliberations for the 2023–2024 vaccination program recommendations [18].

Updated public health and economic analyses are needed to inform ongoing discussions about the health and economic value of the updated COVID-19 vaccines amid the evolving public health and reimbursement landscape. In particular, the availability of a protein-based vaccine in addition to the two mRNA vaccines has the potential to increase vaccine coverage by increasing the choices available to individuals and providers and by potentially expanding vaccine access in underserved or remote communities because of simpler storage requirements. The objective of this study was to estimate the potential population-level health and economic impacts and the cost-effectiveness of including an FDA-authorized, protein-based vaccine as an option for COVID-19 vaccination in the US.

## 2. Materials and Methods

### 2.1. Modeling Approach

We developed a US population model to estimate the health outcomes and economic value associated with the availability of a third vaccine with a different mechanism of action in addition to the two mRNA vaccines for the 2023–2024 COVID-19 vaccination season. The model used a multicohort Markov modeling approach to predict COVID-19 cases and associated costs and outcomes for individuals in the US population meeting the eligibility criteria for a COVID-19 vaccination.

The eligible population was assumed to be all US residents aged 12 years or older, regardless of vaccine or COVID-19 disease history, based on the overlapped current authorization status of all vaccines. The population was subdivided into four age- and risk-based groups on the basis of the likelihood of different levels of severity of COVID-19 disease outcomes. This heterogeneous population represents a mix of those who completed a primary vaccination series, those who received one or more booster vaccines, and those with no prior vaccination. The population also included individuals with a prior confirmed case of COVID-19 and some whose immune status was unknown. High risk for severe COVID-19 illness included immunocompromised adults and adults aged 65 years or older as well as patients with certain medical conditions such as cancer and other chronic diseases [5].

The primary comparators in the model are two different mixes of CDC-recommended vaccines: (1) availability of three 2023–2024 (updated) COVID-19 vaccines (2 mRNA vaccines and 1 protein subunit adjuvanted vaccine (protein-based vaccine)); and (2) availability of only two updated COVID-19 vaccines (2 mRNA vaccines). The primary perspective was that of a third-party payer in the US. This perspective was reflected with US-specific data for epidemiology, vaccines, and healthcare costs; utility loss due to vaccination and due to COVID-19 cases; and mortality due to COVID-19. A societal perspective was also considered in scenario analysis through the inclusion of indirect costs (productivity losses due to vaccination, acute COVID-19 cases, and long COVID).

### 2.2. Model Structure

**Figure 1:**
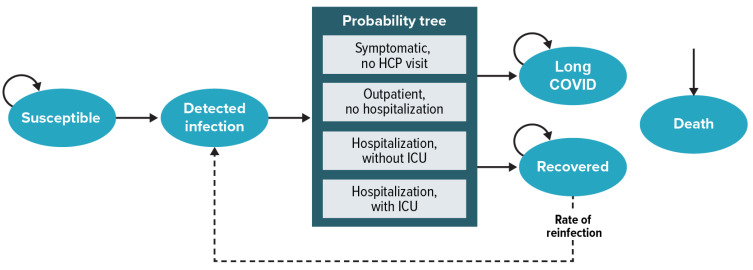
Model structure for an age- and vaccine-specific cohort. COVID-19 = coronavirus disease 2019; ICU = intensive care unit; HCP = healthcare provider. Note: Assumed transition to death from detected infection requiring HCP visit or hospitalization is due to COVID-19, whereas transition to death from all other health states is due to other causes.

The Markov-based structure (Figure 1) used a weekly model cycle length to estimate the number of cases of COVID-19 over a 1-year time horizon for each age and risk group cohort for those not accepting one of the updated COVID-19 vaccines and for those vaccinated with one of the updated COVID-19 vaccines at the time of the program rollout. A probability tree was then used to calculate the distribution of severity levels (no healthcare provider visit, outpatient visit, hospitalization without intensive care unit (ICU) stay, or hospitalization with ICU stay) for all COVID-19 cases for each age and risk group, as illustrated in Figure 1. The model accounted for mortality rates and long COVID rates associated with COVID-19 cases.

For those not receiving an updated COVID-19 vaccine, the weekly probability of a COVID-19 case and the severity distribution were defined by age and risk groups. For those receiving an updated COVID-19 vaccine, the weekly incidence rate and the proportion of cases requiring hospitalization were adjusted on the basis of vaccine effectiveness, taking into account the time elapsed since vaccination and the vaccine effectiveness waning rate.

We estimated economic and population health outcomes for the two vaccination mixes, one with and one without a protein-based vaccine in the mix of available vaccines, by aggregating cohort-level models for those who did and those who did not receive an updated COVID-19 vaccine across all eligible age ranges. We accounted for annual vaccine coverage and market shares for each vaccine mix. Additionally, we estimated the impact on the population outcomes of increasing vaccine coverage for the CDC-recommended vaccination mix, which includes a protein-based vaccine as the third option versus a vaccination mix including only mRNA vaccines.

### 2.3. Model Parameters

The data to parameterize the model were identified from the published literature and other publicly available sources. We prioritized the identification of contemporary US-centric data sources (e.g., CDC, FDA). The epidemiology parameters included estimates of the incidence, distribution of the severity of each COVID-19 case by age and risk groups, and mortality by the severity of COVID-19 without the implementation of a CDC-recommended vaccination program. Vaccine parameters included the effectiveness against symptomatic infection, the effectiveness against hospitalization, corresponding monthly waning rates, and coverage assumptions for the mix with and without a protein-based vaccine. Costs included direct costs per case (by level of severity), vaccine-related costs, costs due to long COVID, and indirect costs due to lost productivity. QALY losses included QALYs lost due to COVID-19 cases (by level of severity), long COVID, vaccination, and COVID-19 death.

#### 2.3.1. Epidemiology

The weekly transitions from susceptible to detected COVID-19 were derived from publicly available data on reported infections in the US from December 2021 through April 2023 to capture the incidence rate since the Omicron strain became dominant in the population, including a majority with prior vaccination or prior COVID-19. The weekly probability of infection in those not receiving a 2023–2024 vaccine (but allowing for prior vaccination or infection) was assumed to be the same for all age and risk groups and was fixed over the model time horizon (Table 1) [19]. In the base case analysis, there were assumed to be no new variants emerging during the 1-year horizon, but variations in the weekly infection rate were evaluated in the scenario analysis.

The severity level distributions by age and risk groups were derived from Li et al. [20] and the Centers for Disease Control and Prevention [21], as were the mortality rates for those experiencing each level of severity (Table 1). We assumed that the distributions of disease severity by age and risk groups identified in the literature represented outcomes for the population not receiving a 2023–2024 vaccine; these parameters also were assumed to remain constant over the 1-year horizon.

**Table 1:** Epidemiology and clinical model parameters.

	12–17 Years	18–64 Years, Low Risk	18–64 Years, High Risk	≥65 Years	Sources and Notes
**Eligible population**					
No. of persons per group (%)	23,803,103 (8.6%)	188,455,975 (67.7%)	12,456,578 (4.5%)	53,624,155 (19.3%)	Derived from [1,22]
**Weekly probability of detected infection (annualized)**					
Base case (post-Omicron dominance (December 2021–April 2023))	0.2341% (11.5%)				Derived from [19]; additional details provided in Table S1
Low (most recent year of available data (May 2022–April 2023))	0.1384% (7.0%)				
High (first year post-Omicron dominance (December 2021–November 2022))	0.2971% (14.3%)				
**COVID-19 severity distribution by highest level of care required**					
Hospitalization, with ICU	0.10%	0.40%	2.10%	2.10%	Derived from [20,21,23]
Hospitalization, without ICU	0.60%	2.40%	10.50%	10.50%	
Outpatient, no hospitalization	31.60%	30.40%	54.20%	54.20%	
Symptomatic, no HCP visit	67.70%	66.80%	33.20%	33.20%	
**COVID-19 mortality probability per event by highest level of care required**					
Hospitalization, with ICU	0.50%	2.20%	5.60%	5.60%	Derived from [21]; differentiation by ICU status not available
Hospitalization, without ICU	0.50%	2.20%	5.60%	5.60%	
Outpatient, no hospitalization	0.00%	0.00%	0.00%	0.00%	
Symptomatic, no HCP visit	0.00%	0.00%	0.00%	0.00%	
**Long COVID proportion of cases (duration)**	7.2% (remainder of model time horizon)				[24]
**Effectiveness against infection, all 2023–2024 COVID-19 vaccines**	56.0%				[5]; assumption
Monthly waning rate	12.8%				Derived from [25]
**Effectiveness against hospitalization, all 2023–2024 COVID-19 vaccines**	73.0%				[5]; assumption
Monthly waning rate	6.0%				Derived from [26]
**Vaccine coverage in eligible population**					
Without updated protein-based COVID-19 vaccine in mix ^a^	7.56%	14.47%	42.44%	42.44%	[27]
With updated protein-based COVID-19 vaccine in mix ^a^	9.51%	18.20%	53.37%	53.37%	Assumption

CDC = Centers for Disease Control and Prevention; COVID-19 = coronavirus disease 2019; FDA = US Food and Drug Administration; HCP = healthcare provider; ICU = intensive care unit; US = United States. ^a^ 2023–2024 COVID-19 vaccine coverage by age based on updated (bivalent) booster dose coverage as of 10 May 2023 [27]. We assumed an increase in overall coverage from 19.4% to 24.4% (five-percentage-point increase) with the updated protein-based COVID-19 vaccine in the mix distributed proportionally across the age and risk groups based on the number of individuals previously vaccinated.

#### 2.3.2. Vaccine Effectiveness and Coverage

The modeled effectiveness of the 2023–2024 COVID-19 vaccines in terms of preventing cases and hospitalizations was based on publicly available data on the relative vaccine effectiveness (VE) of mRNA vaccines [5]. Relative VE represents the effectiveness relative to those with existing immunity rather than absolute effectiveness relative to vaccine-naive individuals. Relative VE was viewed as the most appropriate estimate of vaccine protection for our analysis given that our target population included individuals with varying levels of underlying immunity due to prior vaccination or infection. We assumed that the updated protein-based COVID-19 vaccine had the same effectiveness and durability as the mRNA vaccines (Table 1). We did not account for the second vaccine dose recommended for individuals without prior vaccination, implicitly assuming that the majority of individuals receiving the updated vaccines would be among approximately 90% of the target population with at least one prior COVID-19 vaccination.

Estimates of VE against infection and against hospitalization were based on an interim analysis from 31 August to 31 December 2022 [5]. Monthly waning rates were based on real world data collected for mRNA booster vaccines against the Omicron variant [25,26]. Vaccine coverage by age and risk groups for the mix of two mRNA 2023–2024 vaccines was assumed to be equivalent to the bivalent booster dose coverage as of 10 May 2023 [27]. We assumed that the availability of the protein-based vaccine in the mix of approved vaccines would increase overall coverage from 19.4% to 24.4% (weighted proportionally across the age and risk groups), as shown in Table 1.

#### 2.3.3. Costs and Health-Related Quality of Life

We included US-specific healthcare costs for the updated COVID-19 vaccines (acquisition cost per dose) obtained from publicly available commercial list prices [28]. No administration costs were included in the analysis. US-specific costs for COVID-19-associated healthcare (outpatient and inpatient costs by disease severity) were assigned by the highest level of care required (Table 2). We assumed no direct medical costs associated with adverse events due to vaccination. Lost productivity costs associated with vaccination and with COVID-19 cases were included in societal perspective analyses only.

Health-related quality of life disutilities associated with COVID-19 were combined with durations of impact [29,30] to estimate quality-adjusted life years (QALYs) lost due to COVID-19. COVID-19-related disutility values and durations used in the model are summarized in Table 2. For those in the long COVID health state, the disutility was applied each week for the remainder of the model horizon following a similar assumption from the model published by Sheinson et al. [30].

**Table 2:** Cost and health-related quality of life parameters.

Input Parameter	Baseline Value	Sources and Notes
**Direct costs per case**		
Hospitalization, with ICU	USD 37,429	[31]
Hospitalization, without ICU	USD 13,282	[30]
Outpatient, no hospitalization	USD 282	Derived from [32]
Symptomatic, no HCP visit	USD 0	Assumption
**Average daily cost of lost productivity**		
12–17 years	USD 0	Assumption
18–64 years (low and high risk)	USD 98.95	Derived from data on income by age [33]
≥65 years	USD 25.43	
**COVID-19–related disutility (duration)**		
Hospitalization, with ICU	0.55 (22 days)	[34]
Hospitalization, without ICU	0.30 (17 days)	
Outpatient, no hospitalization	0.19 (10 days)	
Symptomatic, no HCP visit	0.19 (10 days)	
**Vaccine WAC price (CDC cost)**		
Spikevax (Moderna)	USD 128 (USD 81.60)	Adult COVID-19 Vaccine Price List [28]
Comirnaty (Pfizer)	USD 115 (USD 85.10)	
Novavax COVID-19 vaccine, adjuvanted (2023–2024 formula)	USD 130 (USD 58.00)	
**Outcomes due to vaccination**		
Proportion with missed work (duration)	40.9% (0.575 days)	Derived from [35]
Disutility (duration)	0.04 (0.575 days)	Disutility derived from [36]
**Discounted QALYs lost due to COVID-19 death**		
12–17 years	24.8	Derived from [37,38] following the life table method [39] with a 3% discount rate [40]
18–64 years (low and high risk)	17.9	
≥65 years	8.3	
**Long COVID**		
Disutility (duration)	0.19 (up to 1 year)	Assumption
Direct costs per week	USD 51.60	[41]
Total lost productivity	USD 1100	[42]

BLS = Bureau of Labor Statistics; CDC = Centers for Disease Control and Prevention; COVID-19 = coronavirus disease 2019; HCP = healthcare provider; ICU = intensive care unit; QALY = quality-adjusted life year; WAC = wholesale acquisition cost.

### 2.4. Model Outcomes and Analysis

#### 2.4.1. Base Case Analysis

We used the model to predict absolute and incremental health and economic outcomes for the two mixes of CDC-recommended COVID-19 vaccinations. The primary health outcomes for our analysis were the annual numbers of COVID-19 cases, hospitalizations, and deaths due to COVID-19 in the US population. Additional outcomes included direct and indirect costs, QALYs lost, and incremental cost per QALY gained. The differences between the vaccine mix with three vaccines (1 protein-based and 2 mRNA) and the mix with two vaccines (mRNA only) reflect the potential health and economic impacts and the cost-effectiveness in the US population of increasing vaccine coverage through the availability of an updated, protein-based COVID-19 vaccine.

#### 2.4.2. Sensitivity Analyses

Uncertainty analyses are an integral part of economic evaluations [40,43] and are particularly relevant here given the range of unknowns affecting the post-pandemic COVID-19 landscape. We considered the impact of uncertainty on model predictions through scenario analyses constructed around key model parameters and assumptions with significant uncertainty, such as incidence rates, VE, and VE waning rates. Parameter bounds were informed by published literature where available (e.g., 95% confidence intervals) or were approximated using ±20% when additional data were not available. We also performed probabilistic sensitivity analysis on hospitalizations avoided in order to demonstrate the impact of joint, multivariate parameter uncertainties on key model outcomes. Full descriptions of the parameter values and distributions used in the sensitivity analyses can be found in Table S2.

## 3. Results

### 3.1. Base Case Results

For the base case, population-level analysis of the COVID-19 vaccine mix without the updated protein-based vaccine, the model predicted over 31 million COVID-19 cases annually in the US population aged 12 years or older (Table 3). These cases resulted in nearly 1.4 million hospitalizations and over 55,000 COVID-19-related deaths. With this vaccine mix, the model predicted the loss of over 1,042,000 QALYs, primarily due to COVID-19-related deaths, and estimated annual direct costs at over USD 36 billion. Direct costs incurred included USD 7.2 billion in vaccination, USD 3.1 billion in outpatient expenses, USD 23.2 billion due to hospitalizations, and USD 2.9 billion in long COVID costs.

For the CDC-recommended vaccine mix with the updated protein-based vaccine, the analysis predicted that a five-percentage-point increase in coverage due to the availability of a protein-based vaccine option would prevent over 500,000 cases, 66,000 hospitalizations, and 3000 COVID-19-related deaths. Avoiding these clinical outcomes translated to over 42,000 fewer QALYs lost at an additional cost of USD 690 million in direct medical expenses (USD 1.9 billion in additional vaccination costs, partially offset by savings from fewer cases and hospitalizations). For the base case analysis from the perspective of a US third-party payer, our analysis estimated an incremental cost-effectiveness ratio (ICER) of USD 16,141 per QALY gained for the mix of three COVID-19 vaccines (one protein-based and two mRNA) compared with the mix of just two mRNA vaccines.

We also considered the societal perspective by including lost productivity due to vaccination, COVID-19 illness, and long COVID. As expected, lost productivity due to vaccination was predicted to be higher for the mix including the updated protein-based vaccine (USD 234 million higher) due to the increase in coverage. The increases in indirect costs due to vaccination were completely offset by the reductions in lost productivity due to COVID-19 infection (USD 266 million) and long COVID (USD 37 million). From the societal perspective, we estimated an ICER of USD 14,523 per QALY gained for the mix of three COVID-19 vaccines (one protein-based and two mRNA) compared with the mix of just two mRNA vaccines.

Incremental base case results for key outcomes were disaggregated by age category (Table 4). The largest number of incremental cases avoided was in the age 18–64 years, low-risk group, while the number of hospitalizations avoided was estimated to be highest in the group of those aged ≥65 years. Incremental cost-effectiveness ratios generally improved as age increased (e.g., 18–64 years and low risk vs. 12–17 years), and the mix with the protein-based vaccine was estimated to be cost saving for the high-risk group aged 18–64 years and the age ≥65 years group.

### 3.2. Sensitivity Analyses

**Figure 2:**
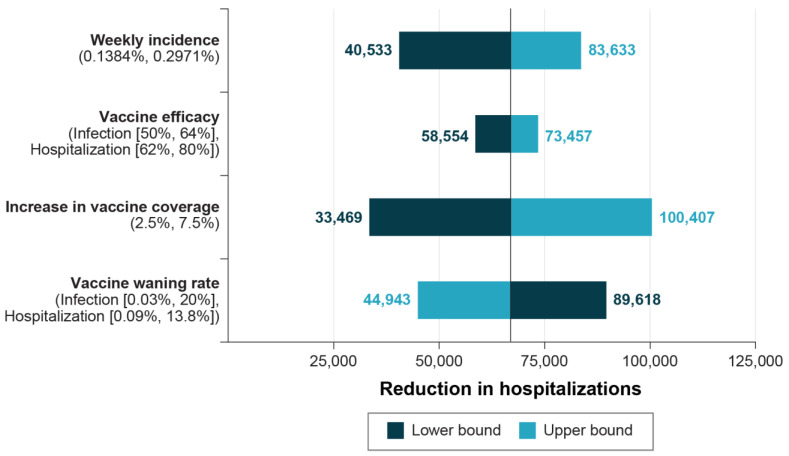
Targeted sensitivity analysis on hospitalizations avoided. Note: Full scenario results are available in Table S3.

We conducted a variety of targeted univariate sensitivity analyses to better understand the impact of variability in key parameters on the potential population-level health and economic impacts of increasing 2023–2024 COVID-19 vaccine coverage through the availability of a protein-based vaccine (Figure 2). Key parameters that influenced the health and economic outcomes included COVID-19 incidence, VE, monthly VE waning rate, and hospitalization costs. Projected COVID-19 incidence had a greater impact on outcomes than did variations in anticipated vaccine effectiveness or the durability of COVID-19 vaccine protection. All scenarios predicted a reduction of at least 33,000 hospitalizations due to the increase in coverage associated with the availability of the updated protein-based COVID-19 vaccine. In a scenario with vaccine effectiveness set to the levels from the placebo-controlled phase three trials (90.4% against infection and 100% against hospitalization [44]), hospitalizations were reduced by over 90,000. Additional incremental health and economic outcomes of interest for all scenarios are presented in Table S3. Of note, while scenarios around the potential increase in vaccine coverage had large impacts on population-level health outcomes, these scenarios did not have a meaningful impact on the ICER (USD 15,588 to USD 17,800 per QALY gained).

**Figure 3:**
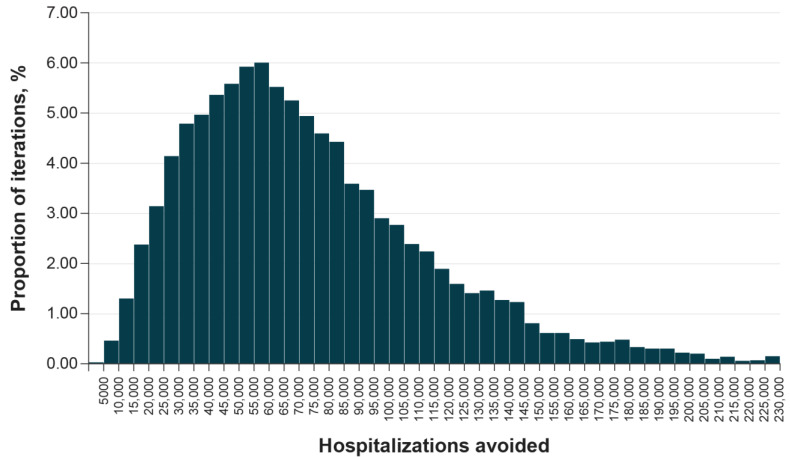
Probabilistic sensitivity analysis on hospitalizations avoided. Note: Probabilistic sensitivity analysis results based on 10,000 iterations. The full list of parameter values and probability distributions is provided in Table S2.

In addition to the targeted univariate sensitivity analyses, we conducted a probabilistic sensitivity analysis to capture the variability in predicted reductions in hospitalizations as model parameters were varied simultaneously. The results of the probabilistic sensitivity analysis for the base case analysis are presented in Figure 3. Overall, the probabilistic mean reduction in hospitalizations (73,781) due to the availability of the updated protein-based COVID-19 vaccine was comparable to the deterministic value (66,938). Over 80% of the probabilistic sensitivity analysis iterations resulted in a reduction in hospitalizations in the range of 25,000 to 110,000, and at least 10,000 hospitalizations were avoided in over 99% of the iterations.

## 4. Discussion

The static, population-based analysis presented in this study estimated the magnitude of the potential reductions in cases, hospitalizations, and deaths from COVID-19 as well as the associated changes in costs and QALYs lost with the availability of a third, protein-based option among the vaccines recommended by the CDC for the 2023–2024 COVID-19 vaccination program. The results showed that an assumed five-percentage-point increase in COVID-19 vaccine coverage for the US population aged 12 years and older attributable to the availability of a protein-based vaccine could reduce the number of cases by 500,000, the number of hospitalizations by 66,000, and the number of deaths by 3000. Assuming commercially listed vaccine prices (USD 115 to USD 130 per dose for all currently authorized or approved 2023–2024 COVID-19 vaccines [28]), the increase in coverage was predicted to result in higher total population healthcare costs despite reductions in the costs associated with the reduced case numbers and severity. The incremental cost-effectiveness of the vaccination program with increased coverage for the overall US population aged 12 years and older from a third-party payer perspective was predicted to be USD 16,141 per QALY gained, with the ICER being more favorable for those aged ≥65 years (cost saving) than for those aged 12–17 years or 18–65 years with low risk (USD 251,338/QALY gained and USD 88,364/QALY gained, respectively).

Our results were driven by the assumption that coverage would increase with the availability of a third vaccine that is protein-based, has a different mechanism of action than mRNA vaccines, and is developed using an approach commonly used for vaccine development for other diseases. The primary reason why coverage might increase with the additional vaccine is that the storage requirements for protein-based vaccines are typically simpler than those for mRNA vaccines, as protein-based vaccines require storage only at regular refrigerator temperatures and not below. This might make such vaccines more readily available to populations living in rural or low-income neighborhoods, where pharmacies or other providers might not have the storage capacity for mRNA vaccines. Higher COVID-19 incidence and greater disease severity have been shown in rural populations [45] and in some racial/ethnic minority populations [9], possibly associated with the lower vaccination rates in those populations.

As in our analysis, many previous cost-effectiveness analyses for the COVID-19 vaccine [15,16] used a population-based approach over a 1-year or longer time horizon with assumptions about potential vaccine coverage. The population-based approach, in contrast with a cohort-based approach, is frequently used in vaccine cost-effectiveness analyses [46] and has added value because it produces estimates of both the cost-effectiveness of an intervention and the annual budget impact of the vaccination program. This can be useful when planning for public health programs and health plans. The model’s 1-year time horizon was selected to align the analysis with the current consensus among major regulatory and public health authorities that COVID-19 vaccines would need to be updated every year to keep up with the evolution of the SARS-CoV-2 virus. However, the shorter time horizon might limit the potential influence of factors such as vaccine durability and long COVID lasting beyond 1 year. Additionally, the uncertainty regarding emerging variants of the virus, their transmissibility and virulence, and the potential impact on vaccine effectiveness make modeling long-term impacts more challenging.

None of the analyses reviewed in a 2023 systematic literature review of COVID-19 vaccination models [16] included the impacts of herd immunity for those not receiving a vaccine, and these impacts were not included in our model. Herd immunity would magnify the benefits of a vaccination program. The input data in the earlier cost-effectiveness analyses were based on (1) early estimates of population incidence and disease severity and (2) the limited treatment options available. The earlier cost-effectiveness analyses all estimated that COVID-19 vaccination was cost-effective at commonly accepted US willingness-to-pay thresholds. However, since those cost-effectiveness analyses were completed, changes have occurred in the annual incidence and severity of COVID-19 due to increases in population immunity to the disease as well as changes in vaccine and treatment availability and prices. These shifts support the added value of the current study estimating the outcomes of the 2023–2024 COVID-19 vaccination program and the potential cost-effectiveness of vaccination in the post-pandemic landscape.

With infectious diseases such as influenza and COVID-19, which are caused by rapidly mutating viruses, the protection afforded against currently circulating viruses by immunity gained through previous infection or from a previous vaccine is likely to decrease over time, as has been shown in studies of COVID-19 since 2020. In particular, the protection against cases of COVID-19 that is offered by the mRNA vaccines has been shown to wane faster than the protection against serious disease (including hospitalization and death) offered by these vaccines [25,47]. There are currently no head-to-head comparative trials of mRNA vaccines and protein-based vaccines that compare the rates of waning protection for the different vaccines. This supports the CDC’s decision to recommend a single 2023–2024 COVID-19 vaccination for authorized age ranges with either an mRNA or a protein-based vaccine designed to better target the currently circulating strains of the virus that causes COVID-19. The success of this program across different age- and risk-based groups in 2023–2024 may influence the decision in subsequent years of whether to continue to recommend an annual cycle with a single targeted shot designed to protect against the COVID-19 virus strains predicted to be circulating. Available studies suggest vaccination may be more cost-effective than many of the therapeutic interventions available in inpatient and outpatient settings [48,49,50]. However, additional analysis is required to fully evaluate the ongoing value of vaccination relative to therapeutic interventions in the post-pandemic setting.

In many ways, the COVID-19 pandemic highlighted the best and the worst of society’s ability to deal with a worldwide event. While there were missteps in the US at the start of the pandemic [51,52], the high points of the response included public–private partnerships and the real-time utilization of decision sciences and data sciences [51,52,53]. In response to the pandemic, governments and other policymakers in the US and around the world worked through public–private partnerships to implement nonpharmaceutical interventions (e.g., masks, social distancing) while accelerating research and regulatory alignment for developing tests, treatments, and vaccines for the SARS-CoV-2 virus. Further, the real time sharing of data and research through expedited channels (e.g., epidemiology, immunology, economic evaluations) provided governments and decision-makers with a means to make vital decisions under intense time pressure and significant uncertainty.

A key strength of our study is its population-based modeling approach, similar to previous COVID-19 analyses, which allowed us to estimate the population-level health outcomes as well as the predicted budget impact and cost-effectiveness of the 2023–2024 COVID-19 vaccination options. In addition, our model includes updated input parameters based on observed incidence data, distributions of disease severity costs, vaccine effectiveness, and costs. One limitation of our study is the unpredictability of COVID-19 virus mutations and the associated impact on the effectiveness of all the updated vaccines in the year following vaccination. This limitation is also observed with annual influenza vaccination, where the effectiveness of the target vaccine varies from year to year. Another limitation is the assumption around the increase in coverage when adding a protein-based vaccine to the mix of CDC-recommended vaccines. While we evaluate the impact of variations in coverage on health outcomes, we do not consider the other potential costs incurred to increase vaccine coverage beyond the additional unit cost of the vaccine (e.g., public health campaigns, increased costs of storage, and wastage to reach underserved areas). The ultra-cold storage requirements for the mRNA vaccines were not considered, although this factor may make protein-based vaccines more attractive for regions where this type of storage poses a barrier to access. The static nature of our model also limits our ability to capture indirect effects of vaccination such as herd immunity or the impact on the rate of SARS-CoV-2 variant mutation. A fourth limitation of our study is the lack of head-to-head studies comparing the effectiveness and durability of protection among different vaccines. Our assumption, based on data from clinical trials, including preclinical studies of the protein-based vaccine, was that they are all similar. We have also included scenario analyses changing these assumptions. To the extent that potential increases in 2023–2024 vaccine coverage occur in previously unvaccinated individuals (who would require two doses of the protein-based vaccine), our analysis may underestimate the costs associated with vaccination. Finally, recent publications suggest vaccines may have a direct impact on the incidence of long COVID, including the potential for therapeutic benefit [54,55]. We conservatively assumed an impact on long COVID strictly by virtue of reducing the number of cases.

## 5. Conclusions

For the 2023–2024 COVID-19 vaccination season, our model estimated that an increase in coverage due to the availability of a protein-based vaccine option could prevent over 500,000 cases, 66,000 hospitalizations, and 3000 COVID-19-related deaths. Avoiding these clinical outcomes translated to over 42,000 fewer QALYs lost across the US population at an ICER of USD 16,141 per QALY gained from a third-party payer perspective. Targeted sensitivity analyses found that the health impact and the economic value of vaccination were most sensitive to COVID-19 incidence and severity in different age and risk groups. Including the protein-based vaccine as an updated COVID-19 vaccine option in the US was predicted to be cost-effective and has the potential to reduce hospitalizations and deaths.

## Figures and Tables

**Table 3:** Base case results.

Summary Results (Total in Target Population)	Mix with Updated Protein-Based Vaccine	Mix without Updated Protein-Based Vaccine	Incremental
Approximate US population	331,893,745	331,893,745	0
Number of eligible individuals	284,481,964	284,481,964	0
Number of individuals receiving vaccination	73,558,971	58,485,411	15,073,560
**Health outcomes**			
COVID-19 cases	30,513,657	31,015,349	−501,692
COVID-19 hospitalizations	1,295,688	1,362,626	−66,938
COVID-19 deaths	51,965	55,284	−3319
Long COVID cases	2,136,565	2,171,882	−35,317
**QALYs lost**			
Vaccine adverse events	1905	1515	390
Outpatient cases	149,149	151,397	−2248
Hospitalizations	21,716	22,849	−1134
COVID-19 deaths	619,468	654,907	−35,439
Long COVID	207,617	211,972	−4355
* Total QALYs lost*	*999,855*	*1,042,641*	*−42,785*
**Direct costs (in millions)**			
Vaccine costs	USD 9205.45	USD 7248.10	USD 1957.35
Outpatient costs	USD 3096.97	USD 3153.00	USD −56.03
Hospitalization costs	USD 22,142.00	USD 23,292.80	USD −1150.80
Long COVID costs	USD 2856.82	USD 2916.74	USD −59.92
* Total direct costs*	*USD 37,301.23*	*USD 36,610.64*	*USD 690.59*
**Indirect costs (lost productivity in millions)**			
Due to vaccination	USD 1144.09	USD 909.64	USD 234.44
Due to COVID-19	USD 14,128.26	USD 14,394.33	USD −266.07
Due to long COVID	USD 2122.33	USD 2159.93	USD −37.60
* Total indirect costs*	*USD 17,394.68*	*USD 17,463.91*	*USD −69.23*
**Third-party payer perspective (direct costs only)**			
Incremental cost per QALY gained (i.e., per QALY losses avoided)	USD 16,141		
**Societal perspective (including indirect costs due to lost productivity)**			
Incremental cost per QALY gained (i.e., per QALY losses avoided)	USD 14,523		

COVID-19 = coronavirus disease 2019; QALY = quality-adjusted life-year; US = United States.

**Table 4:** Base case results disaggregated by age and risk groups.

Age and Risk Groups	Incremental Cases	Incremental Hospitalizations	Incremental Direct Costs (In Millions)	Incremental QALYs Lost	Incremental Cost per QALY Gained
12–17 years	−16,037	−206	USD 59.60	−237	USD 251,338
18–64 years, low risk	−226,705	−11,557	USD 677.69	−7669	USD 88,364
18–64 years, high risk	−47,525	−10,126	USD −8.57	−10,823	Cost saving
≥65 years	−211,425	−45,049	USD −38.13	−24,056	Cost saving

QALY = quality-adjusted life-year.

## Data Availability

All data relevant to this study were obtained from the published literature or other publicly available sources and have been presented in the article and Supplementary Materials.

## Supplementary Materials

The following supporting information can be downloaded at: https://www.mdpi.com/article/10.3390/vaccines12010074/s1, Table S1: Epidemiology Scenarios Based on Published Confirmed COVID-19 Cases by Month; Table S2: Sensitivity Analysis Parameter Values; Table S3: Detailed Scenario Analysis Results.
