# Supplementary material for: Going Forward: Potential Impact of Protein-Based COVID-19 Vaccination Coverage on Population Outcomes and Costs in the United States
Source: Vaccines (Basel). 2024 Jan 12;12(1):74. doi: 10.3390/vaccines12010074 (PMC10819070; doi:10.3390/vaccines12010074)
Supplement: Supplementary file 1 [file vaccines-12-00074-s001.zip › vaccines-2798280-supplementary.pdf]

Going Forward: Potential Impact of Protein-based COVID-19 Vaccination Coverage, Population Outcomes, and Costs in the US

Paret K, Beyhaghi H, Herring WL, Mauskopf J, Shane LG, Rousculp MD

Supplemental Materials

Table S1. Epidemiology Scenarios Based on Published Confirmed COVID-19 Cases by Month

			Scenario		
**Month and year**	**Cumulative cases**	**Monthly cases**	**Most recent year of data**	**Post Omicron**	**1 year post Omicron**
Dec 2020	643,284				
Jan 2021	1,390,234	746,950			
Feb 2021	1,984,537	594,303			
Mar 2021	3,422,248	1,437,711			
Apr 2021	5,151,596	1,729,348			
May 2021	6,343,192	1,191,596			
Jun 2021	7,888,244	1,545,052			
Jul 2021	10,405,391	2,517,147			
Aug 2021	15,434,890	5,029,499			
Sep 2021	23,101,019	7,666,129			
Oct 2021	27,268,025	4,167,006			
Nov 2021	29,494,069	2,226,044			
Dec 2021	31,297,933	1,803,864		1,803,864	1,803,864
Jan 2022	32,716,870	1,418,937		1,418,937	1,418,937
Feb 2022	33,315,233	598,363		598,363	598,363
Mar 2022	33,971,503	656,270		656,270	656,270
Apr 2022	36,252,931	2,281,428		2,281,428	2,281,428
May 2022	40,560,724	4,307,793	4,307,793	4,307,793	4,307,793
Jun 2022	44,642,805	4,082,081	4,082,081	4,082,081	4,082,081
Jul 2022	46,723,945	2,081,140	2,081,140	2,081,140	2,081,140
Aug 2022	50,312,506	3,588,561	3,588,561	3,588,561	3,588,561
Sep 2022	63,354,105	13,041,599	13,041,599	13,041,599	13,041,599
Oct 2022	77,432,340	14,078,235	14,078,235	14,078,235	14,078,235
Nov 2022	79,727,921	2,295,581	2,295,581	2,295,581	2,295,581
Dec 2022	80,541,713	813,792	813,792	813,792	
Jan 2023	82,263,031	1,721,318	1,721,318	1,721,318	
Feb 2023	85,947,586	3,684,555	3,684,555	3,684,555	
Mar 2023	89,048,044	3,100,458	3,100,458	3,100,458	
Apr 2023	92,462,417	3,414,373	3,414,373	3,414,373	
	Cases per week	698,551	449,037	759,398	963,691
	Weekly probability	0.2154%	0.1384%	0.2341%	0.2971%
	Annualized %	10.61%	6.95%	11.48%	14.34%

Note: Data presented in the table are based on monthly cumulative cases since the start of the pandemic [1]. Weekly probability utilizes cases per week divided by the size of the US population.

Table S2. Sensitivity Analysis Parameter Values

Input parameter	Low	Base case	High	Notes
**Weekly incidence**	0.1384%	0.2341%	0.2971%	Derived from [1] in Table S1
**COVID-19 severity by age**				
Hospitalization, 12-17 years	0.55%	0.68%	0.82%	Assumption on uncertainty range (+/-20%); Hospitalization probability reported is combined with and without ICU
Hospitalization, 18-64 years, low risk	2.21%	2.77%	3.32%	
Hospitalization, 18-64 years, high risk	10.02%	12.52%	15.03%	
Hospitalization, ≥ 65 years	10.02%	12.52%	15.03%	
**COVID-19 mortality by age**				
Hospitalization, 12-17 years	0.37%	0.46%	0.56%	Assumption on uncertainty range (+/-20%); Hospitalization probability reported is combined with and without ICU
Hospitalization, 18-64 years, low risk	1.78%	2.22%	2.67%	
Hospitalization, 18-64 years, high risk	4.45%	5.62%	6.75%	
Hospitalization, ≥ 65 years	4.45%	5.62%	6.75%	
**Probability of long COVID**	5.8%	7.2%	9.1%	[2]
**Vaccine parameters (all updated COVID-19 vaccines)**				
Relative efficacy against infection	50%	56%	62%	Confidence intervals around mean for BA.4/BA.5 Bivalent mRNA vaccine [3]
Relative efficacy against hospitalization	64%	73%	80%	
Monthly waning rate of efficacy against infection	0.3%	12.8%	20.0%	Derived from [4]
Monthly waning rate of efficacy against hospitalization	0.9%	6.0%	13.9%	Derived from [5]
**Vaccine coverage overall increase in eligible population**	2.5%	5.0%	7.5%	Assumption

Note: Ranges provided for parameters utilized in sensitivity analyses and is not inclusive of all model parameters.

Table S3. Detailed Scenario Analysis Results

Summary results	Cases avoided	Hospitalizations avoided	Incremental direct costs ($MM)	Incremental QALYs gained	Incremental cost per QALY gained
**Base case**	501,692	66,938	$690.59	42,785	$16,141
**Uncertainty in COVID-19 epidemiology:**					
Decreased incidence	306,325	40,533	$1,190.08	25,751	$46,216
Increased incidence	623,374	83,633	$374.95	53,558	$7,001
**2023-2024 COVID-19 vaccine attributes:**					
Increase in relative effectiveness	555,536	73,457	$565.99	47,059	$12,027
Decrease in relative effectiveness	446,191	58,554	$847.40	37,482	$22,608
Relative increase in durability of protection	956,269	89,618	$212.41	59,483	$3,571
Relative decrease in durability of protection	360,781	44,943	$1,096.24	29,413	$37,270
Optimistic combination[a]	1,060,695	98,451	$38.22	65,500	$583
Pessimistic combination[a]	321,052	39,329	$1,202.39	25,771	$46,656
**Vaccine coverage scenarios:**					
Optimistic increase in coverage	752,538	100,407	$1,000.39	64,178	$15,588
Pessimistic increase in coverage	250,846	33,469	$380.79	21,393	$17,800

Summary results	Cases avoided	Hospitalizations avoided	Incremental direct costs ($MM)	Incremental QALYs gained	Incremental cost per QALY gained
**Additional scenarios of interest:**					
CDC vaccine price [6]	501,692	66,938	-$251.51	42,785	Cost saving
Increased incidence [7]	1,304,339	181,623	-$1,474.60	116,801	Cost saving
Clinical trial efficacy [8]	800,418	92,070	$187.45	60,533	$3,097

[a] Vaccine attribute combinations include efficacy and waning parameter changes (e.g., optimistic = higher relative effectiveness and decrease in waning rate).

References

1. Centers for Disease Control and Prevention. Weekly United States COVID-19 cases and deaths by state. 12 September 2023. https://data.cdc.gov/Case-Surveillance/Weekly-United-States-COVID-19-Cases-and-Deaths-by-/pwn4-m3yp. Accessed 6 October 2023.
2. Montoy JCC. Prevalence of symptoms ≤ 12 months after acute illness, by COVID-19 testing status among adults—United States, December 2020–March 2023. MMWR Morb Mortal Wkly Rep. 2023;72.
3. US Food and Drug Administration. Recommendation for the 2023-2024 formula of COVID-19. 2023. https://www.fda.gov/media/169591/download. Accessed 6 October 2023.
4. Menegale F, Manica M, Zardini A, Guzzetta G, Marziano V, d'Andrea V, et al. Evaluation of waning of SARS-CoV-2 vaccine–induced immunity: a systematic review and meta-analysis. JAMA Netw Open. 2023;6(5):e2310650-e.
5. Wu N, Joyal-Desmarais K, Ribeiro PA, Vieira AM, Stojanovic J, Sanuade C, et al. Long-term effectiveness of COVID-19 vaccines against infections, hospitalisations, and mortality in adults: findings from a rapid living systematic evidence synthesis and meta-analysis up to December, 2022. Lancet Respir Med. 2023;11(5):439-52.
6. Centers for Disease Control and Prevention. Adult COVID-19 vaccine price list. 2023. https://www.cdc.gov/vaccines/programs/vfc/awardees/vaccine-management/price-list/index.html. Accessed 22 September 2023.
7. Prosser L. Economic analysis of COVID-19 vaccination. Presented at Meeting of the Advisory Committee on Immunization Practices, Centers for Disease Control and Prevention; 12 September 2023. Atlanta, GA. https://www.cdc.gov/vaccines/acip/meetings/downloads/slides-2023-09-12/06-COVID-Prosser-508.pdf. Accessed 6 October 2023.
8. Dunkle LM, Kotloff KL, Gay CL, Áñez G, Adelglass JM, Barrat Hernández AQ, et al. Efficacy and safety of NVX-CoV2373 in adults in the United States and Mexico. N Engl J Med. 2022 Feb;386(6):531-43. doi:http://dx.doi.org/10.1056/NEJMoa2116185.
